# Parenteral versus enteral nutrition: effect on serum cytokines and the hepatic expression of mRNA of suppressor of cytokine signaling proteins, insulin-like growth factor-1 and the growth hormone receptor in rodent sepsis

**DOI:** 10.1186/cc5972

**Published:** 2007-07-16

**Authors:** Michael J O'Leary, Aiqun Xue, Christopher J Scarlett, Andre Sevette, Anthony J Kee, Ross C Smith

**Affiliations:** 1 Department of Intensive Care, The St George Hospital, Kogarah, NSW 2217, Australia; 2 Department of Surgery, Royal North Shore Hospital, St Leonards, NSW 2065, Australia

## Abstract

**Introduction:**

Early nutrition is recommended for patients with sepsis, but data are conflicting regarding the optimum route of delivery. Enteral nutrition (EN), compared with parenteral nutrition (PN), results in poorer achievement of nutritional goals but may be associated with fewer infections. Mechanisms underlying differential effects of the feeding route on patient outcomes are not understood, but probably involve the immune system and the anabolic response to nutrients. We studied the effect of nutrition and the route of delivery of nutrition on cytokine profiles, the growth hormone–insulin-like growth factor-1 (IGF-I) axis and a potential mechanism for immune and anabolic system interaction, the suppressors of cytokine signaling (SOCS), in rodents with and without sepsis.

**Methods:**

Male Sprague–Dawley rats were randomized to laparotomy (Sham) or to cecal ligation and puncture (CLP), with postoperative saline infusion (Starve), with EN or with PN for 72 hours. Serum levels of IL-6 and IL-10 were measured by immunoassay, and hepatic expressions of cytokine-inducible SH2-containing protein, SOCS-2, SOCS-3, IGF-I and the growth hormone receptor (GHR) were measured by real-time quantitative PCR.

**Results:**

IL-6 was detectable in all groups, but was only present in all animals receiving CLP-PN. IL-10 was detectable in all but one CLP-PN rat, one CLP-EN rat, approximately 50% of the CLP-Starve rats and no sham-operated rats. Cytokine-inducible SH2-containing protein mRNA was increased in the CLP-EN group compared with the Sham-EN group and the other CLP groups (*P *< 0.05). SOCS-2 mRNA was decreased in CLP-PN rats compared with Sham-PN rats (*P *= 0.07). SOCS-3 mRNA was increased with CLP compared with sham operation (*P *< 0.03). IGF-I mRNA (*P *< 0.05) and GHR mRNA (*P *< 0.03) were greater in the fed CLP animals and in the Sham-PN group compared with the starved rats.

**Conclusion:**

In established sepsis, nutrition and the route of administration of nutrition influences the circulating cytokine patterns and expression of mRNA of SOCS proteins, GHR and IGF-I. The choice of the administration route of nutrition may influence cellular mechanisms that govern the response to hormones and mediators, which further influence the response to nutrients. These findings may be important in the design and analysis of clinical trials of nutritional interventions in sepsis in man.

## Introduction

Early initiation of nutritional support is now considered a standard of care for patients with critical illness in intensive care units. Consensus guidelines recommend the use of enteral nutrition (EN) over parenteral nutrition (PN) unless there is a contraindication to using the gut [1]. Recent studies, however, have shown that it is commonly difficult to achieve adequate nutrition via the enteral route in critically ill patients [2,3]. Meta-analyses of trials comparing EN with PN in critically ill patients have been published [4,5] but interpretation of the results is made difficult by the small sample sizes of individual trials and significant problems with the trial design [5]. The question therefore remains of what is the optimum nutrition regimen for critically ill patients and, in the absence of good quality clinical trial data, clinicians may need to turn to basic science investigations to aid decision-making.

The mechanism by which outcome in critically ill patients might be influenced by the early initiation of nutritional support and the route of delivery of the nutrition is not well understood. Hypotheses favoring EN include prevention of bacterial overgrowth in the stomach or bacterial translocation from the gastrointestinal tract, whereas anabolic effects of the delivered nutrients might favor PN. Any acute effects on patient outcomes, however, are most likely to be mediated through changes in the activity of the immune system. In this regard, the effect of sepsis and the influence of nutrition on tissue protein metabolism and on the functioning of the growth hormone (GH)–insulin-like growth factor-I (IGF-I) axis is of particular interest. While a derangement in the functioning of this axis, termed 'GH resistance', has been implicated as important in the pathogenesis of muscle protein catabolism in critical illness [6], it is now recognized that the activity of anabolic peptides in the GH family and the activity of cytokines are linked through a common cellular receptor [7]. This provides a mechanism whereby changes in the activity of this axis may influence cytokine release, and vice versa.

GH resistance in critical illness is characterized by a rapid and sustained decrease in circulating and tissue concentrations of IGF-I despite elevated circulating levels of GH, the main effector of IGF-I secretion [8]. The mechanism by which GH resistance occurs is not fully understood, but changes both in nutrient availability and in cytokine activation are implicated. Circulating levels of IGF-I and of the insulin-like growth factor binding protein-I (IGFBP-I) are exquisitely sensitive to provision of nutrients, the former being increased and the latter being suppressed by food intake [9]. Hepatic IGFBP-I synthesis is stimulated by the cytokines IL-1, IL-6 and TNFα [10], and circulating IGFBP-I levels are frequently elevated in critically ill patients on intensive care unit admission [11].

Recent work has focused on the potential for a direct interaction between the GH–IGF-I axis and the immune system via the common cellular receptor. The suppressors of cytokine signaling (SOCS) proteins are inhibitors of cytokine and GH signaling via the janus kinase and signal transducer and activator pathway, which appear to inhibit cytokine and GH signaling as part of a classical negative feedback loop [12]. Increased hepatic mRNA of SOCS proteins has been shown to occur transiently in abdominal sepsis and to be temporally associated with the development of GH resistance [13]. In a study employing a rodent model of sepsis – cecal ligation and puncture (CLP) – a relationship was observed between the induction of SOCS and both the presence of sepsis and the administration of PN [14]. Administration of 16 hours of PN was associated with induction of the expression of hepatic mRNA of the SOCS cytokine-inducible SH_2_-containing protein (CIS). This finding suggested a mechanism by which nutrition might modulate both cytokine profiles and the response to anabolic hormones such as GH in sepsis; however, it is not clear whether this observation represents a consequence of an effect of PN, via an effect on cytokine patterns for example, or a consequence of the provision of nutrients *per se*.

We have compared isocaloric and isonitrogenous PN with EN commenced immediately following CLP in rats and continued for 72 hours [15]. We found that PN alone was able to increase hepatic protein synthesis and resulted in improved net skeletal muscle protein metabolism compared with EN. Serum IGF-I was lower in CLP animals administered PN or EN when compared with the matched sham-operated groups. After CLP, PN but not EN was associated with increased IGF-I compared with the levels measured in starved animals. IGFBP-I was increased in CLP animals compared with sham and increased in starved animals compared with those receiving nutrition. PN was associated with the lowest serum IGFBP-I levels in both the CLP and sham-operated groups. We hypothesized that, in sepsis, administration of nutrition and the route of its administration influence hepatic cellular responses to GH by modulation of SOCS proteins, either directly or via differential activation of cytokines. We therefore measured the serum concentrations of a pleiotropic cytokine (IL-6) and an anti-inflammatory (IL-10) cytokine and the expression of mRNA of SOCS proteins, of IGF-I and of the growth hormone receptor (GHR) in hepatic tissue from these animals. These results are reported in the present manuscript.

## Materials and methods

### Experimental design

The Animal Care and Ethics Committee of Royal North Shore Hospital and the University of Technology, Sydney, Australia approved the protocol. Sixty-seven male Sprague–Dawley rats (body weight 180–220g) were received from Gore Hill Animal Research Laboratories (University of Technology, Sydney, Australia) and were housed individually in metabolic cages in a temperature-controlled (23–25°C) and light-controlled (12-hour light/12-hour dark) environment. The animals were initially given access to rat chow and water *ad libitum *for a period of 7 days. Following this acclimatization period, the animals were anesthetized by intraperitoneal injection of ketamine (50 mg/kg body weight) (Ketalar; Parke Davis, Sydney, NSW, Australia) and sodium pentobarbitone (30 mg/kg body weight) (Nembutal; Rhone Merieux, Parramatta, NSW, Australia) and had a catheter aseptically implanted into the right internal jugular vein as described previously [16]. A midline laparotomy was performed and a further catheter was inserted through the anterior wall of the stomach, sutured to the stomach wall and exteriorized through the antero-lateral abdominal wall. This catheter was then subcutaneously tunneled to lie alongside the intravenous line.

The animals were randomized into six groups. At laparotomy, three groups underwent CLP and postoperatively received PN (CLP-PN group, *n *= 12), EN (CLP-EN group, *n *= 13) or a continuous infusion (0.5 ml/hour) of isotonic saline (starvation; CLP-Starve group, *n *= 16). The remaining three groups of animals were subjected to laparotomy only (sham operation; Sham group) and received the same feeding regimen as the CLP animals (Sham-PN group, *n *= 8; Sham-EN group, *n *= 6; and Sham-Starve group, *n *= 12). The PN and EN solutions were identical and provided the daily requirement of energy (1.40 MJ/kg body weight/day), amino acid nitrogen (1.3 g N/kg body weight/day), essential fatty acids, vitamins, minerals and trace elements in a volume equivalent to 230 ml/kg body weight/day [16].

Following anesthesia and surgery, the animals were given 2.5 ml/100 g body weight of 0.9% sodium chloride containing 0.3 mg/kg buprenorphine intraperitoneally to provide fluid resuscitation and analgesia. The rats were then returned to their cages. Oral food (standard rat chow) was removed on the day of operation, but free access to water was continued. Further doses of intraperitoneal fluid and analgesia were administered 24 and 48 hours following operation.

### Cecal ligation and puncture procedure

Following placement of the stomach catheter, in animals randomized to CLP the cecum was identified and tightly ligated at its base with great care taken to ensure that continuity of the bowel was preserved. A 23 G needle was used to puncture the cecum in a single pass through the anterior and posterior walls. The cecum was then gently squeezed to extrude fecal matter. Only one person performed the CLP throughout the entire study to ensure consistency. In sham animals, the cecum was lifted out of the peritoneal cavity, gently squeezed and then returned.

### Procedures at study endpoint

Animals were studied 72 hours following CLP or sham operations. Only animals surviving to this time point could be studied. One animal from the Sham-Starve group died prior to the study endpoint from an unknown cause; all other sham-operated animals survived. Eight CLP-PN rats, five CLP-EN rats and 15 CLP-Starve animals survived. The surviving animals were sacrificed at this time by intravenous injection of a lethal dose of sodium pentabarbitone. Full details of procedures at the time of sacrifice have been previously published [15]. Immediately following sacrifice blood was collected, via cardiac puncture, for measurement of serum levels of IL-6 and IL-10. The abdomen was then opened and the liver was rapidly removed, weighed and flash frozen in liquid nitrogen. The liver was stored at -70°C for subsequent analysis for the expression of mRNA of CIS, SOCS-2, SOCS-3, IGF-I and the GHR.

### Rat IL-6 and IL-10 immunoassays

Serum levels of IL-6 and IL-10 were measured using a Quantikine^® ^Immunoassay system (R&D Systems, Minneapolis, MN, USA) as per the manufacturer's instructions. Briefly, following the addition of 50 μl assay diluent, 50 μl serum (1:1 dilution for IL-6; undiluted for IL-10) was added to the plate and the mixture was incubated for 2 hours at room temperature. The plate was washed five times and then 100 μl conjugate (anti-rat IL-6–horseradish peroxidase; anti-rat IL-10–horseradish peroxidas) was then added and incubated for 2 hours at room temperature. The plate was then washed a further five times and 100 μl substrate solution (equal volumes of hydrogen peroxide and the chromagen tetramethylbenzidine) was added and incubated for a further 30 minutes at room temperature. Finally, 100 μl stop solution (HCl) was added and the absorbance was measured at 450 nm. The minimum limit of detection of the IL-6 assay is 14 pg/ml, and that for IL-10 is <10 pg/ml.

### Measurement of mRNA for SOCS proteins, IGF-I and GHR

The specific primers used for real-time quantitative RT-PCR for targeting mRNA expression values were designed with the assistance of the PRIMER 3 software [17]. The primers were: SOCS2, 5'-GCG TGA GCT CAG TCA AAC AG-3' and 5'-CCC GGC TGA TGT CTT AAC AG-3'; SOCS3, 5'-CCT CAA GAC CTT CAG CTC CA-3' and 5'-CGG TTA CGG CAC TCC AGT AG-3'; CIS, 5'-GCT TGT CGA GAC CTC GAA TC-3' and 5'-CAG GAT CTG GGC TGT CAC TC-3'; IGF-1, 5'-TCA GTT CGT GTG TGG ACC AAG-3' and 5'-TCA CAG CTC CGG AAG CAA C-3'; GHR, 5'-ATC TTT GGC GGG TGT TCT TA-3' and 5'-TAG CTG GTG TAG CCC CAC TT-3'.

Two micrograms of total RNA treated with DNase I (Sigma, St Louis, MO, USA) was used for the RT reaction, with the cDNA stored at -20°C until use. Real-time quantitative RT-PCR was performed using the iCycler iQ system (BioRad, Hercules, CA, USA) employing SYBR Green I fluorescence (Sigma) according to the manufacturer's instructions. Amplification of all mRNAs was performed in duplicate in a PCR 96-well reaction plate (BioRad). The following experimental run protocol was used. cDNA was denatured at 95°C for 5 minutes to activate the Hot-start *Taq *DNA polymerase. The amplification and quantification program was repeated 40 times (95°C for 20 s, 60°C for 1 min, 72°C for 30 s, with a single fluorescence measurement).

After the PCR a melting curve was constructed by increasing the temperature from 55°C to 95°C at a heating rate of 0.5°C/10 seconds with continuous fluorescence measurements. The PCR efficiency (*E*) and the cycle threshold (CT) for each sample was determined using iCycle software (BioRad). The mRNA expression of SOCS2, SOCS3, CIS, IGF-1 and GHR was defined as the mean normalized gene expression (MNE) difference in target gene expression relative to the 'housekeeping gene' 18S rRNA using the following equation [18]: MNE = [(*E*_ref_)^CTref,mean^]/[(*E*_target_)^CTtarget,mean^].

### Statistical analysis

Statistical evaluation of data was performed using analysis of variance with Tukey's test *post hoc *by Instat GraphPad version 5.02 (GraphPad Software, Inc., San Diego, CA, USA). Cytokine measurements below the lower limit of detection of the assays were allocated an arbitrary value of 1 ng/ml to permit intergroup statistical analysis. Differences detected between groups were considered significant at *P *< 0.05.

## Results

### Serum levels of IL-6 and IL-10

**Figure 1:**
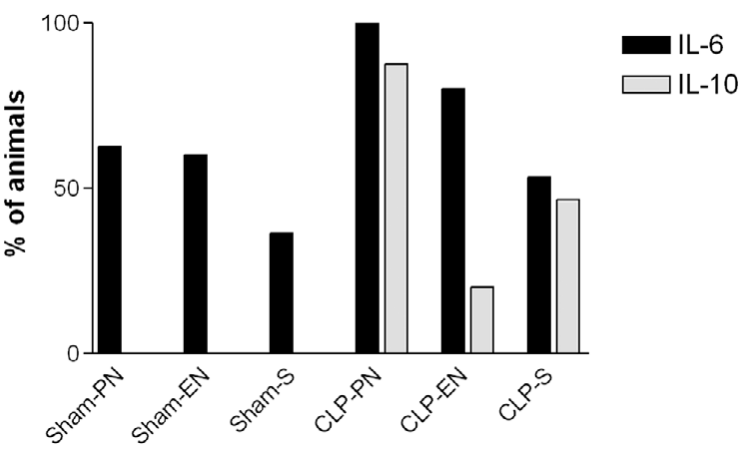
Serum levels of IL-6 and IL-10. Percentage of animals in each of the experimental groups that had serum levels of IL-6 and IL-10 measurable above the lower limits of detection of the assays (IL-6, 14 pg/ml; IL-10, <10 pg/ml). Sham, sham operation; PN, parenteral nutrition; EN, enteral nutrition; S, starvation; CLP, cecal ligation and puncture.

**Figure 2:**
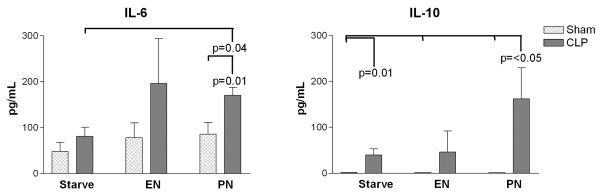
Results of serum cytokine assays. Graphs illustrate serum levels of IL-6 and IL-10, measured 72 hours after sham operation (Sham) or cecal ligation and puncture (CLP) in rats with postoperative infusion of saline (Starve), enteral nutrition (EN) or parenteral nutrition (PN). Bars and error bars represent mean values and standard error of the mean. Significant differences between groups indicated where *P *< 0.05.

Circulating IL-6 was measurable in animals from each of the experimental groups, but only the group receiving PN following CLP had measurable levels in all animals. In each of the other groups a number of animals had levels below the lower limit of detection of the assay (Figure 1). Animals with undetectable levels of IL-6 were more frequent in the Starve groups than in those receiving nutrition. The only differences for IL-6 that attained statistical significance were in animals receiving PN following CLP, where IL-6 levels were greater compared both with starvation following CLP and with PN following the sham operation (Figure 2).

Levels of IL-10 were below the lower limit of detection of the assay in all animals in the sham-operated groups and in all but one of the animals receiving EN following CLP, whereas all but one of the CLP-PN animals had measurable levels of circulating IL-10 (Figure 1). The levels of IL-10 measured in the CLP-PN group were significantly greater than those measured in all sham-operated groups (Figure 2).

### Hepatic expression of mRNA for CIS, SOCS-2, SOCS-3, IGF-I and GHR

**Figure 3:**
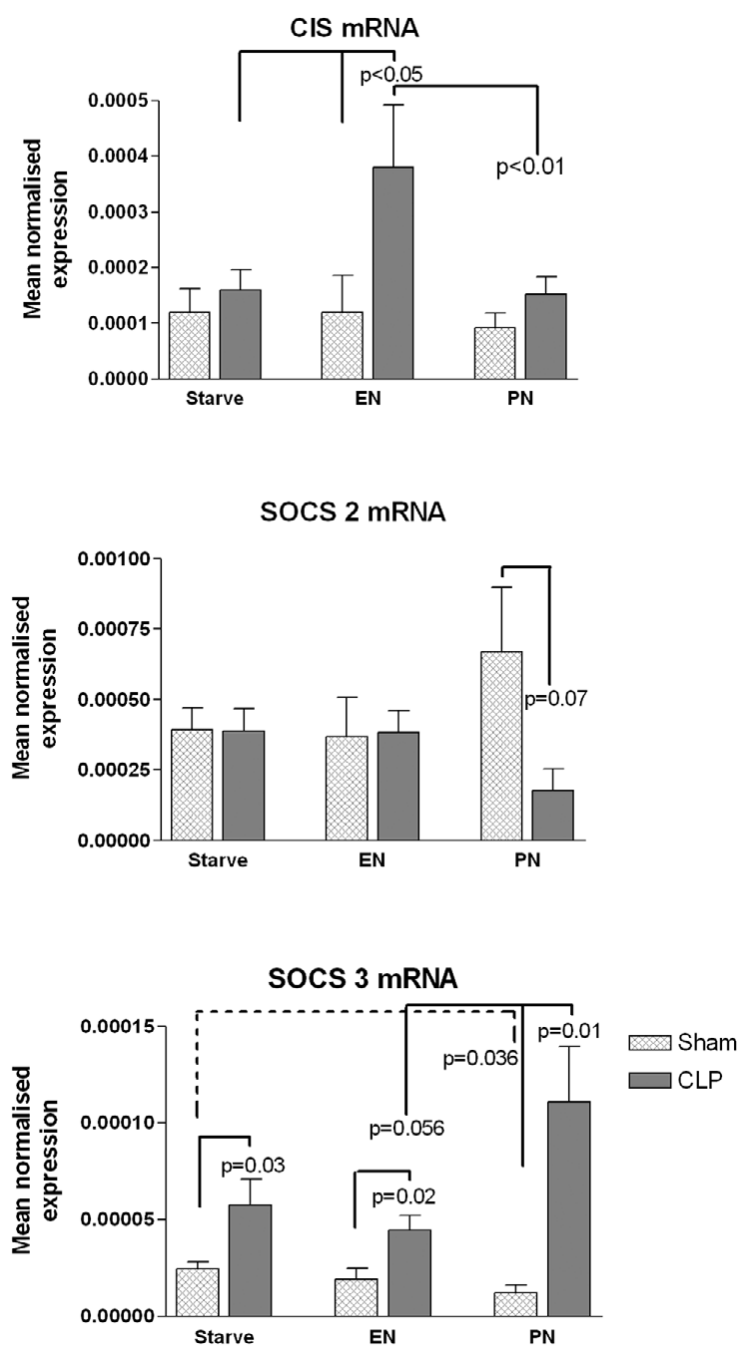
PCR measurements for cytokine-inducible SH_2_-containing protein, suppressors of cytokine signaling-2 and suppressors of cytokine signaling-3. Bars represent the mean expression of mRNA of cytokine-inducible SH_2_-containing protein (CIS), suppressors of cytokine signaling (SOCS)-2 and SOCS-3, normalized to 18S rRNA (mean normalized expression), measured in the liver from rats 72 hours after laparotomy only (Sham) or cecal ligation and puncture (CLP), with postoperative saline infusion (Starve), enteral nutrition (EN) or parenteral nutrition (PN). Error bars represent standard error of the mean. Significant differences between groups indicated where *P *< 0.05.

The MNE of mRNA for CIS was significantly increased in CLP-EN rats compared with Sham-EN animals and compared with animals from the other CLP groups (Figure 3). The MNE of mRNA for SOCS-2 was decreased in CLP-PN animals compared with Sham-PN animals, but was otherwise not different between the groups. The SOCS-3 mRNA MNE was significantly increased in all CLP animals when compared with sham animals from the matched feeding groups. In addition, the MNE was greater in CLP-PN animals compared with CLP-EN animals (*P *= 0.056). In the sham-operated animals, the MNE of mRNA for SOCS-3 was significantly lower in animals receiving PN compared with starvation animals.

**Figure 4:**
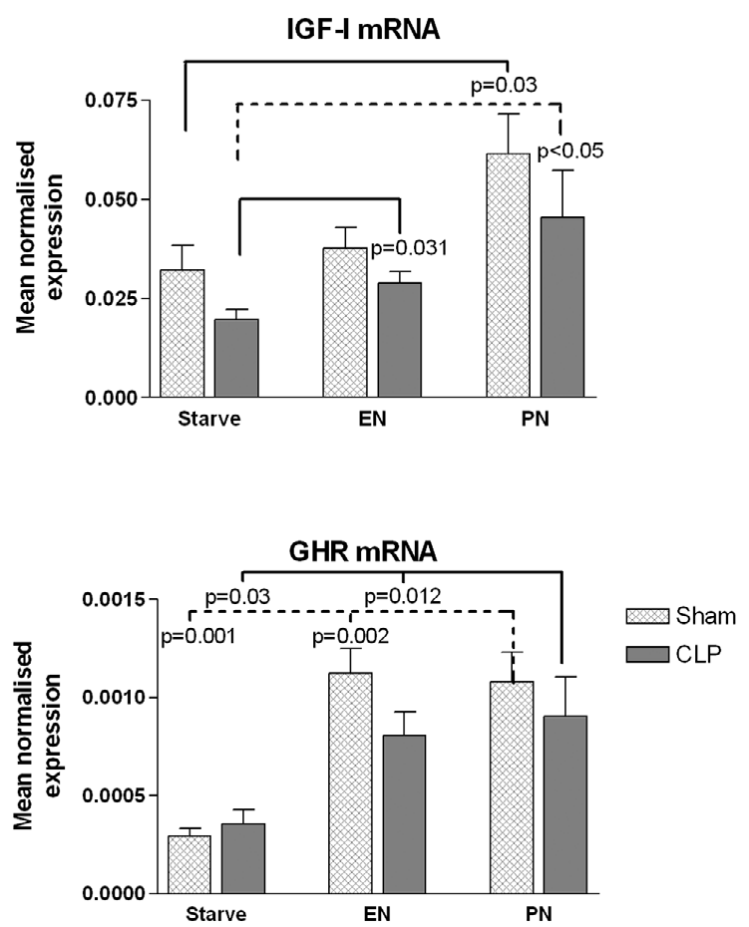
PCR measurements of insulin-like growth factor-1 and the growth hormone receptor. Bars represent the mean expression of mRNA of insulin-like growth factor-1 (IGF-I) and growth hormone receptor (GHR), normalized to 18S rRNA (mean normalized expression), measured in the liver from rats 72 hours after laparotomy only (Sham) or cecal ligation and puncture (CLP), with postoperative saline infusion (Starve), enteral nutrition (EN) or parenteral nutrition (PN). Error bars represent standard error of the mean. Significant differences between groups indicated where *P *< 0.05.

The MNE of mRNA for IGF-I was in general increased by feeding compared with starvation, significant differences being observed between both the CLP-PN group and the CLP-EN group and CLP-Starve group, and between Sham-PN rats and Sham-Starve rats (Figure 4).

The MNE of mRNA for the GHR was increased by feeding compared with starvation for both CLP and sham-operated animals, but there was no difference comparing PN with EN in either of the surgical groups (Figure 4).

## Discussion

In this study we have shown that the use of EN compared with PN in rats with abdominal sepsis can influence serum levels of IL-6 and IL-10. The route of administration of nutrition also influenced the expression of mRNA of SOCS proteins in the liver. CIS was increased in sepsis by EN and SOCS-2 in sham operation by PN, whereas SOCS-3 was increased with PN after CLP and decreased with PN after sham operation. Nutrition increased the expression of mRNA of both IGF-I and the GHR, while these were not affected by sepsis. These results support a potential effect of nutrition and the route of administration of nutrition on the activity of the GH–IGF-I axis that may be mediated by cytokine production and by alterations in intracellular signaling mechanisms involving the SOCS proteins.

A number of studies in both animals and man show differences in immune system function in association with the administration of PN compared with EN [19-22]. These differences are considered to be driven principally by changes at the level of the mucosa of the gastrointestinal tract. In mice, the presence or absence of nutrients within the gut lumen has a major influence on the size and function of the gut mucosal immune system. PN is associated with a rapid fall in lymphocyte cell counts and a change in cell profiles in gut-associated lymphoid tissue; this profile change is related to decreased levels of the Th_2 _cytokines IL-4 and IL-10 [23]. These changes appear to be important since, compared with chow feed, PN in animals is associated with enhanced transport of endotoxin across the gut [24] and with increased bacterial recovery from mesenteric lymph nodes [25].

Studies in man, however, are conflicting. In human volunteers receiving PN versus EN, the administration of endotoxin was associated with higher temperature, higher C-reactive protein, higher epinephrine and higher TNFα responses in the PN group in one study [26] – whereas in another study the responses to endotoxin were essentially comparable, albeit with a reduced IL-6 response [27]. Notwithstanding these changes and their theoretical importance, controlled trials in man have repeatedly demonstrated an increase in infections in patients administered PN compared with EN or no nutrition [4,5], a clinical observation that lends weight to differential effects of the two routes of feeding on immune function. As might be expected, 72 hours following CLP or sham operation we found a wide scatter of serum concentrations of IL-6 and IL-10. Nonetheless, there appeared to be differences in the pattern of cytokine concentrations related both to the presence or absence of sepsis and to the nutritional management of the animals, with recovery of circulating IL-6 and IL-10 being more frequent in animals with sepsis administered PN. Furthermore, if these differences are explained by the effect of absence of EN on the gastrointestinal tract, it is possible that a more prolonged period of PN, as frequently used in patients, might have produced a more marked differential in cytokine recovery between PN and EN animals.

In the clinical management of critically ill patients, balanced against concerns that use of PN predisposes to deleterious immunological changes are the risks associated with failure or delay in provision of nutrition when attempted via the enteral route. We have found that PN is superior to EN in increasing hepatic and muscle protein synthesis and circulating levels of IGF-I [15]. PN also resulted in significantly lower IGFBP-I levels compared with EN in septic animals, despite the greater recovery of IL-6 and IL-10 in PN-fed animals with sepsis. Our observations that PN was more efficacious, in comparison with EN, in influencing circulating IGF-I and IGFBP-I levels are in contrast to another rodent study comparing PN and EN in sepsis [28]. In that study, however, nutrition was commenced 48 hours prior to the septic insult, which is not comparable with the usual situation in patients with sepsis. Furthermore, the feeds administered were not identical.

In the present study we found increased hepatic mRNA of IGF-I in association with PN. In addition, hepatic mRNA of the GHR was increased with both PN and EN. These findings suggest that nutrition is an important stimulant to the synthesis of GHRs and thus IGF-I. We failed to demonstrate significant differences between septic and sham-operated animals in expression of mRNA of the GHR or IGF-I, nutritional differences appearing to be of more importance. The effect of sepsis on GHRs remains uncertain. After CLP both increased specific binding of GH to the liver [29] and reduced expression of hepatic mRNA of GHRs have been demonstrated [14]. Reduced receptor binding and mRNA was found following endotoxin challenge [30], whilst unchanged GHR mRNA was demonstrated after fecal agar pellet implantation [13]. Although the pathophysiology of GH resistance in sepsis is still not fully understood, the current consensus view is that low circulating concentrations of IGF-I indicate a defect in GH signal transduction that may occur either at the level of the GHR or be associated with a change in the intracellular signaling pathway for GH.

The induction of SOCS proteins by hormones and/or cytokines has been hypothesized to inhibit GH signaling by a negative feedback loop involving the janus kinase and signal transducer and activator pathway [12]. Yumet and colleagues [13] have recently shown in rats with abdominal sepsis that total GHR numbers are unchanged, with the impaired IGF-I response to GH being temporally related to a defect in STAT5 activation and increased SOCS mRNA expression. SOCS-1 and CIS expression were increased 4 hours following induction of sepsis, but by 24 hours were no different from measurements in sham-operated animals – whereas SOCS-3 expression remained elevated at 24 hours. The mechanism of the increase in SOCS expression in abdominal sepsis is unknown, and the authors of the study commented that the time course observed was consistent with that produced by endotoxin and inflammatory cytokines. Hepatic mRNA of CIS, SOCS-2 and SOCS-3 is transiently increased following endotoxin administration [31], while SOCS-1 expression and SOCS-3 expression were found to be increased 24 hours following CLP [14]. We now demonstrate continued induction of SOCS-3 expression at 72 hours after CLP, associated with reduced hepatic expression of mRNA of IGF-I and reduced serum IGF-I concentrations. SOCS-3 may be of particular importance in the mechanism of GH resistance in sepsis.

In light of the previous study that demonstrated an effect of PN on CIS expression [14], we were particularly interested in the possibility that nutrition, and possibly the route of feeding, might influence SOCS expression and thus GH resistance. We found greater SOCS-3 expression with PN compared with EN in sepsis, whereas in sham-operated animals the SOCS-3 expression was lowest in those given PN. In animals with sepsis, the CIS expression was increased by EN, and SOCS-2 expression was greater in sham-operated animals than septic animals given PN. As seen in the prior study, however, SOCS-2 expression was not affected by sepsis [14]. These differences in SOCS expression appeared independent of the induction of mRNA of the GHR or of IGF-I. Our hypothesis is that differential effects of PN versus EN on circulating levels of cytokines can explain these differences, but it seems unlikely that these effects are modulated via changes in number of GHRs. While differences in cytokine levels were observed, any mechanistic influence of these on SOCS expression cannot be determined from the present study.

We recognize that there may be problems with interpretation of the results of the present study. Although we attempted to limit the size of the ligated area of the cecum and to ensure bowel continuity was maintained, if generalized peritonitis occurred after CLP the resultant ileus and intestinal ischemia may have made nutrition by the enteral route impossible. Nonetheless, EN is recommended in clinical management guidelines for a number of conditions where intraabdominal sepsis may occur, and is said to be tolerated even in the setting of the ileus [32]. The model is therefore clinically relevant to human abdominal sepsis, in which EN use may be considered. The model has the advantage over previous studies that nutrition was commenced after the septic insult was initiated, as would most probably occur in patients, and that the EN and PN were identical. Furthermore, the measurements were made at a single time point 72 hours following operation. Cytokine levels change in a dynamic way after CLP, with the most pronounced changes occurring transiently well prior to 72 hours. Our experimental model precluded repeated blood sampling, but it is our contention that the differences at 72 hours are more likely to be influenced by the nutritional manipulations than would changes at earlier time points.

## Conclusion

We found that nutrition and the route of nutrition in sepsis differentially influence circulating cytokine profiles and the expression of mRNA of SOCS proteins, of the GHR and of IGF-I. The present study demonstrates that the choice of nutrition route in sepsis may influence cellular mechanisms that govern the response to hormones and mediators, which further influence the response to nutrients themselves. Although our results may be heavily influenced by the design of the experiment and the experimental model, the complex interactions illustrated should be considered in the design of future trials of nutritional management in patients with sepsis.

## Key messages

• The route of administration of nutrition (parenteral versus enteral) in sepsis influences circulating levels of IL-6 and IL-10 in rodents.

• Sepsis and the route of nutrition influence mRNA of SOCS proteins, CIS and SOCS-2, whereas SOCS-3 mRNA is increased in sepsis independent of nutrition.

• Provision of nutrition increased mRNA of the GHR and of IGF-I.

• These findings suggest that provision of nutrition and the route of delivery of nutrition in sepsis can influence circulatory and cellular mechanisms that link cytokines and the GH–IGF-I axis

## Abbreviations

CIS = cytokine-inducible SH_2_-containing protein; CLP = cecal ligation and puncture; CT = cycle threshold; *E *= PCR efficiency; EN = enteral nutrition; GH = growth hormone; GHR = growth hormone receptor; IGF-I = insulin-like growth factor-1; IGFBP-I = insulin-like growth factor binding protein-1; IL = interleukin; MNE = mean normalized expression; PCR = polymerase chain reaction; PN = parenteral nutrition; RT = reverse transcriptase; SOCS = suppressors of cytokine signaling; TNF = tumor necrosis factor.

## Competing interests

MJO'L has received honoraria from Baxter Australia Pty Ltd, and from Fresenius Pharmatel Pty Ltd. The other authors declare that they have no competing interests.

## Authors' contributions

MJO'L and RCS conceived of and designed the study. All authors participated in the animal handling and procedures. CJS carried out the immunoassays and AX performed the PCR studies. MJO'L performed the statistical analysis. MJO'L, AX and CJS helped to draft the manuscript. All authors read and approved the final manuscript.
